# Editorial: Understanding & Improving Performance in Strength Sports

**DOI:** 10.3389/fspor.2022.807941

**Published:** 2022-02-16

**Authors:** James Steele, Justin Keogh, Jeremy P. Loenneke

**Affiliations:** ^1^Faculty of Sport, Health, and Social Sciences, Southampton Solent University, Southampton, United Kingdom; ^2^Faculty of Health Sciences and Medicine, Bond University, Gold Coast, QLD, Australia; ^3^Health, Exercise Science and Recreation Management, University of Mississippi, Oxford, MS, United States

**Keywords:** strength, power, strongman, CrossFit, weightlifting

The “Iron Game,” as the culture of strength exercise and sports has come to be known, has a long rich history.^1^ Resistance training practices have existed in varied forms for millennia. Indeed, as early as 480 BC Greek soldiers engaged in a form of strength training, referred to as calisthenics [from *kalós* ($\kappa \overset{´}{\alpha }\lambda \lambda$oς), which means “beautiful,” and *sthenos* ($\sigma \theta \tilde{\varepsilon }\nu$oς), meaning “strength”], using their bodyweight to provide resistance during exercise. Further, endeavors of strength have existed as sports from the earliest of Olympic games. It is from these origins though that modern strength sports emerged, such as Highland Games and modern Olympic weightlifting which appeared around the Nineteen century. The development of strength has been a source of scientific interest for over a century now (Kraemer et al., 2017; Nuzzo, 2021a). Many technological developments in strength equipment have been used in scientific research, and also for displays of strength (Nuzzo, 2021b). Nowadays, a plethora of different sports fall under the category of “strength sports” with Olympic weightlifting, powerlifting, and strongman being among the most popular. Despite the name “strength sports” implying “strength” as the main performance outcome, many strength sports test a multitude of physiological capabilities in combination with maximal strength (e.g., anaerobic capacity, power, muscular endurance). The popularity of strength sports has been on the rise in the past decade, with competitions and participants reaching all-time highs. Participation in some of the biggest powerlifting federations in the USA has increased substantially in recent years. Similar to powerlifting, the sport of strongman has also been rapidly growing with its “World's Strongest Man” event reaching approximately 220 million views in 2019. Yet, despite the rise of strength sports in popularity both in terms of competition and recreational participation in their training methods, there is currently very little scientific evidence available specifically regarding these sports particularly from the perspective of training and performance. Hence, this Research Topic was developed to encourage exploration of this area. It contains research examining both biomechanical elements of strength sports performance considering how this may impact training approaches for its development, and mixed methods investigation into training practices for strength sports athletes.

Hindle et al. investigated the biomechanics of the strongman yoke walk in experienced male and female strongman competitors. Their findings highlighted that the yoke walk was characterized by flexion of the hip and slight to neutral flexion of the knee at heel strike, slight to neutral extension of the hip and flexion of the knee at toe-off and moderate hip and knee range of motion (ROM), with high stride rate and stance duration, and short stride length. Such information may better inform athletes and strength and conditioning coaches to: provide athletes with recommendation on how to perform the yoke walk based on the technique used by experienced strongman athletes; better prescribe exercises to target training adaptations required for improved yoke walk performance; and better coach the yoke walk as a training tool for non-strongman athletes.

Werner et al. explored strength sports athletes performing their sport; this time weightlifters and the changes in kinematics of the clean movement across heavier and lighter barbell loads. They applied different approaches including principal component analysis (PCA) to determine what characteristics are identified best with either approach. The first PCA (PCA^trial^) analyzed variance among time-normed waveforms compiled from subjects and trials; the second PCA (PCA^posture^) analyzed postural positions compiled over time, subjects and trials. They concluded that: first, barbell load affects specific aspects of the movement pattern of the clean; and second, the PCA^trial^ approach is better suited for detecting deviations from a mean motion trajectory and its results are easier to interpret; the PCA^posture^ approach reveals coordination patterns and facilitates comparisons of postural speeds and accelerations. They suggest that their combined data visualizations including stick-figure animations might aid athletes or coaches and provide an objective tool for technique assessments. Such an approach contrasts with classical video analysis, where only the individual technique of one athlete at a time can be assessed, or where individual athletes are compared to each other, but cannot be compared to whole groups.

Several studies from the same group also examined elements of technique and the “sticking regions” of both the bench press and squat lifts with potential implications for both athletes performance, and also recreational lifters. Regarding the bench press, Larsen, Gomo, et al. explored the effects of grip width on the joint, barbell kinematics, and horizontal kinetics, analyzed in tandem with the muscle activation around the sticking region in the 1-RM barbell bench press. Further, they explored barbell, joint kinematics, joint kinetics of hip, knee, and ankle in tandem with myoelectric activity around the sticking region (Larsen, Kristiansen, van den Tillaar, et al.), and the roles of barbell placement (van den Tillaar et al.) and stance width (Larsen, Kristiansen., Helms, et al.). These studies highlight the biomechanical and technique-based approaches that enable lifting the greatest loads and target activation of certain musculature within the bench press and squat.

The deadlift is a common exercise for both athletes and recreational lifters, as well as being one of the three powerlifts. Further, it has become increasing popular to combine other implements with free weights for training purposes. Thus, Andersen et al. explored the acute effects of elastic bands to either add resistance, or provide assistance, during the deadlift. Their findings suggest athletes and practitioners wanting to producing as much force as possible when performing the deadlift, should combine free weights with elastic bands, instead of using free weights only. Such differences between either resisted or assisted deadlifts may make them more or less appropriate depending on specific context.

Lastly, and one of the few multi-study mixed methods papers in sport and exercise sciences, Androulakis-Korakakis et al. explored the emerging concept of the “minimum effective training dose” (METD). This included an interview study with elite powerlifting athletes and highly experienced powerlifting coaches, an interview and survey study with powerlifting coaches and powerlifting athletes of all levels, two training intervention studies with intermediate-advanced powerlifting athletes and a survey study with competitive powerlifting athletes of different levels. They suggested that powerlifting athletes looking to train with a METD approach can still expect to gain meaningful strength improvements for 6–12 weeks by performing ~3–6 working sets of 1–5 repetitions each week, with these sets spread across 1–3 sessions per week per powerlift, using loads above 80% 1RM at an RPE of 7.5–9.5. This might be desirable during periods of limited time availability, deloads, as well as a potential competition preparation tool. Further, doing so may be a useful strategy to manage fatigue, injury risk (among powerlifters who already train frequently with high loads and reduced volume), and thereby, enhance longevity in the sport.

In summary, we hope that this Research Topic contributes valuable evidence and practical guidance for strength sports athletes and coaches, as well as recreationally trained individuals looking to utilize such methods for their own training. Though still in its infancy, the diverse range of exploratory studies and methodological approaches employed here should hopefully spark further interest and research in the field. We anticipate a bright future for the evidence base in strength sports.

## Author Contributions

All authors listed have made a substantial, direct, and intellectual contribution to the work and approved it for publication.

## Conflict of Interest

The authors declare that the research was conducted in the absence of any commercial or financial relationships that could be construed as a potential conflict of interest.

## Publisher's Note

All claims expressed in this article are solely those of the authors and do not necessarily represent those of their affiliated organizations, or those of the publisher, the editors and the reviewers. Any product that may be evaluated in this article, or claim that may be made by its manufacturer, is not guaranteed or endorsed by the publisher.

## Footnotes

^1^Iron Game History: The Journal of Physical Culture (https://starkcenter.org/research-2/iron-game-history/) is one such outlet that curates much of this history.
